# Evaluation of Prediction Models for Identifying Malignancy in Pulmonary Nodules Detected via Low-Dose Computed Tomography

**DOI:** 10.1001/jamanetworkopen.2019.21221

**Published:** 2020-02-14

**Authors:** Sandra González Maldonado, Stefan Delorme, Anika Hüsing, Erna Motsch, Hans-Ulrich Kauczor, Claus-Peter Heussel, Rudolf Kaaks

**Affiliations:** 1 Division of Cancer Epidemiology, German Cancer Research Center, Heidelberg, Germany; 2 Translational Lung Research Center Heidelberg, German Center for Lung Research, Heidelberg, Germany; 3 Division of Radiology, German Cancer Research Center, Heidelberg, Germany; 4 Department of Diagnostic and Interventional Radiology, University Hospital Heidelberg, Heidelberg, Germany; 5 Department of Diagnostic and Interventional Radiology with Nuclear Medicine, Thoraxklinik-Heidelberg GmbH, Heidelberg, Germany

## Abstract

**Importance:**

Malignancy prediction models based on participant-related characteristics and imaging parameters from low-dose computed tomography (CT) may improve decision-making regarding nodule management and diagnosis in lung cancer screening.

**Objective:**

To externally validate 5 malignancy prediction models that were developed in screening settings, compared with 3 models that were developed in clinical settings, in terms of discrimination and absolute risk calibration among participants in the German Lung Cancer Screening Intervention trial.

**Design, Setting, and Participants:**

In this population-based diagnostic study, malignancy probabilities were estimated by applying 8 prediction models to data from 1159 participants in the intervention arm of the Lung Cancer Screening Intervention trial, a randomized clinical trial conducted from October 23, 2007, to April 30, 2016, with ongoing follow-up. This analysis considers end points up to 1 year after individuals’ last screening visit. Inclusion criteria for participants were at least 1 noncalcified pulmonary nodule detected on any of 5 annual screening visits, receiving a lung cancer diagnosis within the active screening phase of the Lung Cancer Screening Intervention trial, and an unequivocal identification of the malignant nodules. Data analysis was performed from February 1, 2019, through December 5, 2019.

**Interventions:**

Five annual rounds of low-dose multislice CT.

**Main Outcomes and Measures:**

Discrimination ability and calibration of malignancy probabilities estimated by 5 models developed in data from screening studies (4 Pan-Canadian Early Detection of Lung Cancer Study [PanCan] models using a parsimonious approach including nodule spiculation [PanCan-1b] or a comprehensive approach including nodule spiculation [PanCan-2b], and PanCan-2b replacing the nodule diameter variable with mean diameter [PanCan-MD] or volume [PanCan-VOL], as well as a model developed by the UK Lung Cancer Screening trial) and 3 models developed in clinical settings (US Department of Veterans Affairs, Mayo Clinic, and Peking University People’s Hospital).

**Results:**

A total of 1159 participants (median [range] age, 57.63 [50.34-71.89] years; 763 [65.8%] men) with 3903 pulmonary nodules were included in this study. For nodules detected in the prevalence round of CT, the PanCan models showed excellent discrimination (PanCan-1b: area under the curve [AUC], 0.93 [95% CI, 0.87-0.99]; PanCan-2b: AUC, 0.94 [95% CI, 0.89-0.99]; PanCan-MD: AUC, 0.94 [95% CI, 0.91-0.98]; PanCan-VOL: AUC, 0.94 [95% CI, 0.90-0.98]), and all of the screening models except PanCan-MD and PanCan-VOL showed acceptable calibration (PanCan-1b: Spiegelhalter *z* = −1.081; *P* = .28; PanCan-2b: Spiegelhalter *z* = 0.436; *P* = .67; PanCan-MD: Spiegelhalter *z* = 3.888; *P* < .001; PanCan-VOL: Spiegelhalter *z* = 1.978; *P* = .05; UK Lung Cancer Screening trial: Spiegelhalter *z* = −1.076; *P* = .28), whereas the other models showed worse discrimination and calibration, from an AUC of 0.58 (95% CI, 0.46-0.70) for the UK Lung Cancer Screening trial model to an AUC of 0.89 (95% CI, 0.82-0.97) for the Mayo Clinic model.

**Conclusions and Relevance:**

This diagnostic study found that PanCan models showed excellent discrimination and calibration in prevalence screenings, confirming their ability to improve nodule management in screening settings, although calibration to nodules detected in follow-up scans should be improved. The models developed by the Mayo Clinic, Peking University People’s Hospital, Department of Veterans Affairs, and UK Lung Cancer Screening Trial did not perform as well.

## Key Points

**Question:**

Are nodule malignancy prediction models developed in screening contexts superior to those developed in clinical contexts in terms of discrimination and calibration?

**Findings:**

In this diagnostic study of 1159 participants from the German Lung Cancer Screening Intervention trial, models originally developed using data from the Pan-Canadian Early Detection of Lung Cancer Study showed excellent discrimination and calibration compared with models developed in clinical contexts when applied to prevalence screening low-dose computed tomography images.

**Meaning:**

These findings confirmed the good performance and transferability of the Pan-Canadian Early Detection of Lung Cancer Study models in population screening settings, especially for nodules detected on prevalence screen.

## Teaser

This diagnostic study examines the efficacy of 8 models for predicting malignancy in pulmonary nodules detected via low-dose computed tomography.

## Introduction

The US National Lung Cancer Screening Trial (NLST)^1^ and other randomized clinical trials in Europe^2,3,4,5^ have shown that low-dose (LD) computed tomography (CT) is a viable screening tool for reducing lung cancer mortality among long-term smokers. However, screening entails harms in the form of false-positive screening test referrals and follow-up diagnostics. The efficiency of cancer screening using CT depends in part on optimized criteria for reporting and managing lung nodules; therefore, radiologic and oncologic expert societies have developed guidelines^6,7,8,9,10^ for using nodule characteristics as predictors of malignancy and as decision-making indicators to guide further diagnostic work.

For nodules detected by CT for which there is an absence of information on nodule growth, several research groups have developed statistical models to determine the likelihood of malignancy based on radiologic features and patient-related attributes, such as age, sex, smoking history, presence of emphysema or other respiratory diseases, and personal or family history of cancer. Initial models were based on data from clinical settings, such as at the Mayo Clinic,^11^ US Department of Veterans Affairs (VA) clinics,^12^ or Peking University People’s Hospital (PKUPH).^13^ However, these models were fitted to data from patients with high pretest probability of malignancy, focused on larger, mostly solid and often solitary nodules detected incidentally or in symptomatic individuals, and may overestimate malignancy risk for nodules detected in screening contexts.^14^

Malignancy prediction models were first developed in a screening setting at Brock University, Toronto, Canada, using data from prevalence screenings in the Pan-Canadian Early Detection of Lung Cancer Study (PanCan) LDCT screening trial.^15^ Initial PanCan models^15^ (sometimes also referred to as *Brock models*) were generated using either a parsimonious (hereafter, *PanCan-1*) or a comprehensive (hereafter, *PanCan-2*) approach for variable selection without (PanCan-1a and PanCan-2a) or with (PanCan-1b and PanCan-2b) nodule spiculation among the radiologic variables. The more comprehensive model including spiculation, PanCan-2b, was recently updated by replacing nodule diameter with multidimensional measurements: mean diameter (PanCan-MD) or volume (PanCan-VOL).^16^ Independently of PanCan, a new model was developed recently in the context of the UK Lung Cancer Screening (UKLS) trial^17^ using nodule volume.

The PanCan models have been externally validated for discrimination in the NLST^16,18^ and Danish Lung Cancer Screening Trial,^19^ but they have not been externally validated for calibration. The recent UKLS model^17^ has not been externally validated, to our knowledge. Using data from the German Lung Cancer Screening Intervention (LUSI) trial,^5,20^ we evaluated the earlier and more recent versions of the PanCan models, as well as the UKLS model, in terms of discrimination ability, calibration, and operational performance (eg, sensitivity, specificity, and positive predictive values). For comparison, we also present findings for the Mayo Clinic, VA, and PKUPH models, as well as for pulmonary nodules first observed in follow-up (ie, incidence) screenings.

## Methods

### Study Design and Participants

The LUSI trial^5,20^ is a screening trial among adults aged 50 to 69 years with a history of heavy smoking (defined as ≥25 years of smoking ≥15 cigarettes per day or ≥30 years smoking ≥10 cigarettes per day and ≤10 years since smoking cessation) randomized into a screening intervention group, which included an LD multislice CT scan at time of randomization and 4 annual follow-up screening examinations, and a control group with no intervention. Participants were recruited as a random sample from population registries in Heidelberg, Germany, and surrounding areas and were assigned to the CT screening group or the control group. All participants provided written informed consent. The LUSI trial was approved by the ethics committee of the University of Heidelberg and the German Federal Office for Radiation Protection. This analysis of LUSI trial data is covered under the original ethical board approval per regulations of the ethics committee of the University of Heidelberg. This study followed the Standards for Reporting of Diagnostic Accuracy (STARD) reporting guideline for diagnostic studies.

### Image Acquisition and Reading

Computed tomography scans were obtained using a Toshiba 16-row scanner from October 23, 2007, to December 31, 2009, or a Siemens 128-row scanner from March 18, 2010, until the end of the screening phase of the LUSI trial on May 25, 2016. We used Median computer-aided detection software (Median Technologies) to read CT images, and nodule measures were derived, with largest diameter given in millimeters, perpendicular diameter given in millimeters, and volume given in millimeters-cubed. Perifissural nodules with oval or triangular shape and/or smooth delineations were excluded under the assumption that they were lymphatic nodules. Further data collected for each CT scan included nodule identifier, location, type (ie, solid or subsolid), shape and border characteristics (eg, presence of spiculation, translucence), and calcification. Presence of emphysema was determined on CT images using a densitometry method.^21^

### Nodule Evaluation and Management

All CT images were evaluated by 2 trained chest radiologists (including S.D.), and decisions were made based on nodule size and growth (in cases of nodules that had previously been recorded) (eTable 1 in the Supplement). Participants with a lung cancer diagnosis made later than 1 year after the last CT scan or for whom the malignant nodule(s) could not be identified in earlier CT images were excluded.

### Spirometry Data

In all study participants, spirometry was performed using MasterScreen IOS (VIASYS Healthcare) to determine 1-second forced expiratory volume (FEV_1_) and forced vital capacity (FVC), and FEV_1_ to FVC ratios were calculated from the largest FEV_1_ and FVC values recorded in any 1 of 2 repeated assessments. Detailed descriptions of imaging, nodule, and lung function assessments are given in the eAppendix in the Supplement.

### Statistical Analysis

All nodules detected during screening were analyzed using data from the LDCT image in which they were first seen. For participant-related characteristics, we used the Mann-Whitney U test for differences in continuous data and the χ^2^ test or Fisher exact test for categorical data, as appropriate. For differences in nodule-specific characteristics, we used mixed-effects logistic regression with participant as the random effect.^22^ We applied 8 models from 6 published studies to our data to calculate the probability of nodule malignancy (eTable 2 in the Supplement): 4 PanCan models (PanCan-1b^15^: parsimonious model including spiculation; PanCan-2b^15^: full model including spiculation; PanCan-MD^16^: PanCan-2b with the mean of the largest and perpendicular diameters as nodule size; and PanCan-VOL^16^: PanCan-2b with nodule volume as nodule size), the recently developed UKLS model^17^; and the models developed in VA clincs,^12^ the Mayo Clinic,^11^ and PKUPH clinics.^13^

We explored associations of radiologic parameters and participant-related characteristics with risk of nodule malignancy by fitting multivariable logistic regression models via generalized estimating equations using prevalence and incidence screening rounds combined and accounting for the correlation structure of multiple pulmonary nodules per individual on the LUSI data. Model selection was based on the quasi–Akaike Information Criterion^23^ (eAppendix in the Supplement).

For each selected model, the ability to discriminate nodules by malignancy status was evaluated through cluster-adjusted receiver operating characteristic curves and the corresponding area underneath the curve (AUC).^24^ Given the correlation structure of nodules within participants, sensitivity, specificity, and positive and negative predictive values were estimated using generalized estimating equations.^25,26,27^ For comparison, we also estimated the discrimination capacity of models fitted on LUSI databased on a larger set of variables using cluster bootstrapping (*B* = 1000).^24^ Model calibration was assessed by examining observed vs predicted nodule malignancy rates by category of nodule size (<5 mm, 5 to <8 mm, 8 to 10 mm, or >10 mm; as used in the LUSI CT evaluation algorithm) as well as by deciles of predicted risk. The Hosmer-Lemeshow goodness of fit test^28^ was used to examine the fit between predicted and observed malignancy probabilities across deciles of predicted risk, and Brier scores (BS) and Spiegelhalter *z* test were used to assess the overall deviance of risk predictions estimated by models vs observed rates.^29^ Hosmer-Lemeshow goodness of fit and χ^2^ for independence *P* values were 1-sided, and all other *P* values were 2-sided. Statistical significance was set at .05.

All analyses were performed using R statistical software for version 3.5.1 (R Project for Statistical Computing), with packages gee, Hmisc, lme4, MuMIn, rms, pROC, and ROCR, as well as the clusteredROC function. Data analysis was performed from February 1, 2019 to December 5, 2019.

## Results

### Study Participants and Pulmonary Nodule Findings

During 5 screening rounds, 1182 participants in the LUSI LDCT arm showed at least 1 noncalcified pulmonary nodule. A total of 62 participants were diagnosed with lung cancer up to 12 months after their last LDCT screening participation: 56 cancers detected via screening and 6 interval cancers.^5^ For 54 of 56 cancers detected via screening, malignancy was linked to 1 (51 participants) or more (3 participants) nodules. For 2 cancers detected via screening without nodules, the decision of referral to further diagnostic work was based on other pulmonary abnormalities. For 2 of 6 interval cases, no nodules were observed on LDCT screenings; for the remaining 4 cases with nonsuspicious nodules, no information was available to unequivocally link any of these to malignancy. Nodules with benign-appearing calcification and individuals with lung cancer whose lung tumors could not be linked back to a specific nodule were excluded from statistical analyses.

After removing participants with a lung cancer diagnosis made later than 1 year after the last CT scan or for whom the malignant nodule(s) could not be identified in earlier CT images, data was available for 3903 pulmonary nodules from 1159 individuals (median [range] age, 57.63 [50.34-71.89] years; 763 [65.8%] men) (eFigure 1 in the Supplement). Detailed nodule counts by diameter, type (solid vs subsolid), malignancy status, and screening round of first observation (prevalence or incidence) are presented in eTable 3 in the Supplement. Of 3903 nodules observed, 2883 nodules (73.9%; 32 of these [1.1%] identified as malignant) were first seen during the prevalence screening, whereas 1020 nodules (26.1%; 31 of these [3.0%] identified as malignant), were first observed in 1 of 4 incidence screenings. Irrespective of screening round, more than 70% of malignant nodules (prevalence round: 25 of 32 nodules [78.1%]; incidence rounds: 25 of 31 nodules [80.6%]) had a diameter of 8 mm or greater. For these nodules 8 mm or greater, the malignancy rate was higher among nodules first observed in the prevalence round (12.8% [95% CI, 8.6%-18.5%]) than among those first observed in the incidence round (8.6% [95% CI, 5.7%-12.5%]); conversely, mean malignancy rate was higher for nodules smaller than 8 mm first observed in incidence rounds (0.8% [95% CI, 0.3%-1.9%]) than those first observed in the prevalence round (0.3% [95% CI, 0.1%- 0.6%]).

Irrespective of screening round of first detection, compared with benign nodules, malignant nodules were significantly more often located in the left upper lobe (561 nodules [14.6%] vs 23 nodules [36.5%]; *P* < .001), were larger in terms of diameter (median [range], 4.8 [2.2-65.6] mm vs 11.6 [3.5-107.7] mm; *P* < .001) and volume (median [range], 41.4 [5.0-28947] mm^3^ vs 357.1 [19.3-17466] mm^3^; *P* < .001) and more often had spiculated borders (193 nodules [5.0%] vs 23 nodules [36.5%]; *P* < .001) (Table 1). Compared with patients with benign nodules, patients with malignant nodules were statistically significantly more likely to be older (median [range] age, 57.38 [50.34-71.89] years vs 59.88 [51.90-69.98] years; *P* < .001), have emphysema (474 patients [42.9%] vs 35 patients [64.8%]; *P* = .002), and have lower FEV_1_ (median [range], 2.88 [0.66-6.11] L vs 2.66 [1.26-4.24] L; *P* = .009); whereas sex, smoking status at randomization, smoking duration and intensity, self-reported history of extrathoracic cancer, years since smoking cessation, presence of asthma or bronchitis, and FVC showed no significant association with malignancy diagnosis in the LUSI data (Table 2). Data on family history of cancer were not available in our data set.

**Table 1.** Nodule Characteristics by Malignancy Status and Screening Round

Characteristic	First Seen on the Prevalence Round (n = 2883)			First Seen on Any Incidence Round (n = 1020)			First Seen on Any Round (n = 3903)		
	Benign (n = 2851)	Malignant (n = 32)	*P* Value^a^	Benign (n = 989)	Malignant (n = 31)	*P* Value^a^	Benign (n = 3840)	Malignant (n = 63)	*P* Value^a^
Diameter, median (range), mm	4.3 (2.30-64.2)	10.6 (3.9-107.7)	<.001	5.90 (2.2-65.6)	11.60 (3.5-38.7)		4.8 (2.2-65.6)	11.6 (3.5-107.7)	<.001
Mean diameter, median (range), mm	3.7 (1.9-35.7)	9.3 (3.7-93.7)	<.001	4.95 (2-51.9)	9.45 (2.7-29.9)		4.0 (1.9-51.9)	9.4 (2.75-93.7)	<.001
Volume, median (range), mm^3^	33.7 (5-8960.3)	384.9 (19-17466)	<.001	63.2 (8-28947.9)	355.9 (21.1-7726.8)	.09	41.4 (5.0-28947)	357.1 (19.3-17466)	<.001
Type, No. (%)									
Solid	2561 (89.8)	30 (93.8)	.01	862 (87.2)	28 (90.3)	.62	3423 (89.1)	58 (92.1)	.09
Subsolid	290 (10.2)	2 (6.2)		127 (12.8)	3 (9.7)		417 (10.9)	5 (7.9)	
Location, No. (%)									
Right upper	715 (25.1)	9 (28.1)	.03	260 (26.3)	7 (22.6)	<.001	975 (25.4)	16 (25.4)	<.001
Right middle	278 (9.8)	1 (3.1)		98 (9.9)	1 (3.2)		376 (9.8)	2 (3.2)	
Right lower	682 (23.9)	9 (28.1)		217 (21.9)	4 (12.9)		899 (23.4)	13 (20.6)	
Left upper	438 (15.4)	9 (28.1)		123 (12.4)	14 (45.2)		561 (14.6)	23 (36.5)	
Left lower	596 (20.9)	4 (12.5)		251 (25.4)	4 (12.9)		847 (22.1)	8 (12.7)	
Lingula	111 (3.9)	0		33 (3.3)	1 (3.2)		144 (3.8)	1 (1.6)	
Unclear	31 (1.1)	0		7 (0.7)	0		38 (1.0)	0	
Nodule shape, No. (%)									
Spiculated	79 (2.8)	13 (40.6)	<.001	114 (11.5)	10 (32.3)	.74	193 (5.0)	23 (36.5)	<.001
Nonspiculated	2277(79.9)	11 (34.4)		619 (62.6)	14 (45.2)		2896 (75.4)	25 (39.7)	
Unclear	495 (17.4)	8 (25.0)		256 (25.9)	7 (22.6)		751 (19.6)	15 (23.8)	
Calcification, No. (%)									
Noncalcified	2785 (97.7)	30 (96.9)	.86	943 (95.2)	29 (93.5)	.29	3728 (97.1)	60 (95.2)	.83
Unclear	66 (2.3)	1 (3.1)		46 (4.7)	2 (6.5)		112 (2.9)	3 (4.8)	
Nodules/image, median (range)	4 (1-22)	2 (1-16)	.32	3 (1-22)	2 (1-20)	.32	4 (1-22)	2 (1-20)	.20

^a^ Derived from a χ^2^ test for the difference in deviance of 2 mixed-effects logistic models: both with participant as random effect and 1 additionally having the corresponding nodule characteristic as fixed effect.

**Table 2.** Participant Characteristics at Randomization

Characteristic	Patients, No. (%)			*P* Value^a^
	Overall (N = 1159)	With Benign Nodules (n = 1105)	With Malignant Nodules (n = 54)	
Age, median (range), y	57.63 (50.34-71.89)	57.38 (50.34-71.89)	59.88 (51.90-69.98)	<.001
Sex				
Women	396 (34.2)	379 (34.3)	17 (31.5)	
Men	763 (65.8)	726 (65.7)	37 (68.5)	
Smoking status at randomization				
Current	718 (61.9)	685 (62.0)	33 (61.1)	>.99
Former	441 (38.1)	420 (38.0)	21 (38.9)	
Smoking duration, median (range), y	37.50 (27.50-52.50)	37.50 (27.50-52.50)	37.50 (27.50-52.50)	.06
Smoking intensity, median (range), cigarettes/d, No.	22.50 (12.50-62.50)	22.50 (12.50-62.50)	22.50 (12.50-62.50)	.31
History of cancer^b^				
Yes	3 (0.3)	2 (0.2)	1 (1.0)	
No	1156 (99.7)	1103 (99.8)	53 (98.1)	
History of cancer^c^				
Yes	109 (9.4)	103 (9.3)	6 (11.1)	.63
No	1050 (90.6)	1002 (90.7)	48 (88.9)	
Time since quitting smoking, median (range), y	4 (0.04-13.00)	4 (0.04-13)	8 (0.30-13)	.16
Emphysema				
Yes	509(43.9)	474 (42.9)	35 (64.8)	.002
No	650 (56.1)	631 (57.1)	19 (35.2)	
Asthma				
Yes	47 (4.1)	47 (4.3)	0	.16
No	1112 (95.9)	1058 (95.7)	54 (100)	
Bronchitis				
Yes	209 (18.0)	201 (18.2)	8 (14.8)	.72
No	950 (82.0)	904 (81.8)	46 (85.2)	
FEV_1_, median (range), L	2.87 (0.66-6.11)	2.88 (0.66-6.11)	2.66 (1.26-4.24)	.009
FVC, median (range), L	3.75 (0.33-6.78)	3.75 (0.33-6.78)	3.45 (1.96-5.69)	.08

Abbreviations: FEV_1_, 1-second forced expiratory volume; FVC, forced vital capacity.

^a^ Derived using a Mann-Whitney U test for differences in continuous variables or a χ^2^ test or Fisher exact test for differences in the distribution of categories between the 2 groups, depending on cell counts.

^b^ Excludes lung cancer or cancer within the past 5 years.

^c^ Excludes lung cancer.

### Evaluation of Predictive Models

For nodules detected at the participants’ first screening (prevalence round), all PanCan models showed high discrimination accuracy. Among the PanCan variants, the comprehensive PanCan-2b model achieved only marginally better discrimination (AUC, 0.94 [95% CI, 0.89-0.99]) than the parsimonious PanCan-1b model (AUC, 0.93 [95% CI, 0.87-0.99]), and there was no improvement in discrimination with the use of volume in the PanCan-VOL model (AUC, 0.90 [95% CI, 0.90-0.98]) or 2-dimensional perpendicular mean diameter in the PanCan-MD model (AUC, 0.94 [95% CI, 0.91-0.98]). The discrimination by UKLS was poor (AUC, 0.58 [95% CI, 0.46-0.70]). A reduced UKLS model (eTable 2 in the Supplement) that ignored variables with definitions or prevalence markedly differing from LUSI performed better in our data (prevalence round: AUC, 0.79 [95% CI, 0.68-0.89]; incidence rounds: AUC, 0.60 [95% CI, 0.49-0.70]) compared with the original version. For comparison, the models originally developed in clinical settings showed moderately good discrimination, (VA : AUC, 0.84 [95% CI, 0.76-0.92]; Mayo Clinic: AUC, 0.89 [95% CI, 0.82-0.97]; PKUPH: AUC, 0.87 [95% CI, 0.79-0.995]) (Figure).

**Figure.**
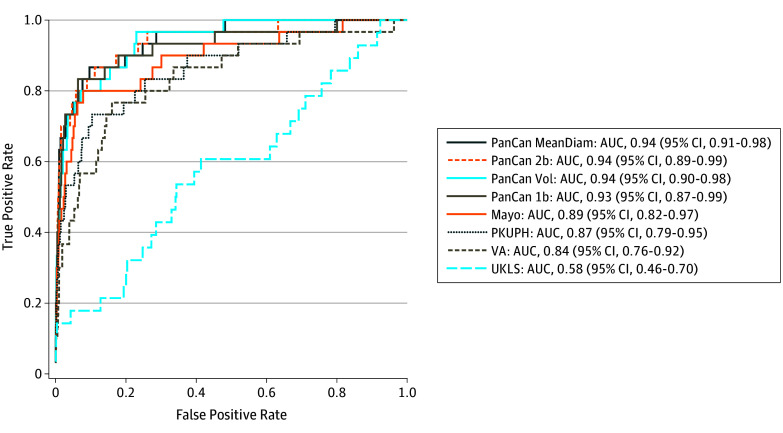
Receiver Operating Characteristic Curves Among Nodules First Seen in the Prevalence Round of Screening AUC indicates area under the curve; Mayo, model developed by the Mayo Clinic; PanCan 1b, Pan-Canadian Early Detection of Lung Cancer Study (PanCan) model using a parsimonious approach including spiculation; PanCan 2b, PanCan model using a comprehensive approach including spiculation; PanCan MeanDiam, PanCan 2b model replacing nodule diameter with mean diameter (calculated as [largest nodule diameter + perpendicular diameter]/2); PanCan Vol, PanCan-2b model replacing nodule diameter with volume; PKUPH, model developed by the Peking University People’s Hospital; UKLS, UK Lung Cancer Screening trial model; VA, model developed by the US Department of Veterans Affairs.

Compared with the prevalence round, all models (except for UKLS) showed useful, although reduced, discrimination when applied to nodules first noticed in any of the incidence rounds (PanCan-1b: AUC, 0.93 [95% CI, 0.87-0.99]; PanCan-2b: AUC, 0.94 [95% CI, 0.89-0.99]; PanCan-MD: AUC, 0.94 [95% CI, 0.91-0.98]; PanCan-VOL: AUC, 0.94 [95% CI, 0.90-0.98]; UKLS: AUC, 0.58 [95% CI, 0.46-0.70]; VA: AUC, 0.84 [95% CI, 0.76-0.92]; Mayo Clinic: AUC, 0.89 [95% CI, 0.82-0.97]; PKUPH: AUC, 0.87 [95% CI, 0.79-0.97]) (eFigure 2 in the Supplement). This difference may be explained by reduced variability in nodule size (Table 1; eTable 3 in the Supplement).

Regarding model calibration, for nodules detected at the prevalence screen, visual inspection of predicted absolute probabilities of malignancy and observed malignancy rates within categories defined by nodule size suggested best agreement for PanCan-1b (Table 3; eTable 4 in the Supplement). Additional comparisons within deciles of the predicted probability scores (eTable 5 and eTable 6 in the Supplement) combined with Hosmer-Lemeshow (HL) tests for deviance between predicted and observed rates showed acceptable calibration for PanCan-1b (HL = 7.71; *P* = .56), PanCan-2b (HL = 7.23; *P* = .61), and PanCan-VOL (HL = 10.89; *P* = .28) but not for PanCan-MD (HL = 30.53; *P* < .001) or UKLS (HL = 158.99; *P* < .001) (eTable 7 in the Supplement). Alternatively, using BSs and Spiegelhalter *z* tests, acceptable calibration was found for PanCan-1b (BS = 0.009; Spiegelhalter *z* = −1.081; *P* = .28), PanCan-2b (BS = 0.009; Spiegelhalter *z* = 0.436; *P* = .67), and UKLS (BS = 0.012; Spiegelhalter *z* = −1.076; P = .28) but not for PanCan-VOL (BS = 0.009; Spiegelhalter *z* = 1.978; *P* = .05) or PanCan-MD (BS = 0.009, Spiegelhalter *z* = 3.888; *P* < .001) (eTable 7 in the Supplement). Malignancy probabilities estimated by models developed in clinical contexts were all strongly overestimated (VA: Spiegelhalter *z* = −25.24; *P* < .001; Mayo Clinic: Spiegelhalter *z* = −12.63; *P* < .001; PKUPH: Spiegelhalter *z* = −19.35; *P* < .001) (Table 3) and were poorly calibrated according to all tests performed (VA: BS = 0.063; HL = 774.38; *P* < .001; Mayo Clinic: BS = 0.014; HL = 162.32; *P* < .001; PKUPH: BS = 0.094; HL = 1119.9; *P* < .001). None of the models showed acceptable calibration on nodules first observed in incidence screens.

**Table 3.** Observed Absolute Risk for Nodule Malignancy vs Predicted Model Estimates by Nodule Size During Prevalence Screening^a^

Nodule Size, mm^b^	Total Nodules, No.	Malignant Nodules, No.	Observed Malignancy Rate, %	Predicted Malignancy Rate, %							
				PanCan-1b^c^	PanCan-2b^c^	PanCan-MD^c^	PanCan-VOL^c^	UKLS^c^	VA^d^	Mayo^d^	PKUPH^d^
<5	1820	1	0.05	0.2	0.2	<0.1	0.1	1.5	20.7	5.9	24.5
5 to <8	868	6	0.69	1.3	0.9	0.4	0.7	1.8	23.6	7.4	27.6
8 to 10	110	7	6.36	4.6	3.5	2.3	3.1	2.2	29.7	10.6	34.8
>10	85	18	21.18	18.6	14.7	12.9	11.9	6.8	48.9	29.1	52.3
Total	2883	32	1.11	1.2	1.0	0.6	0.7	1.8	22.8	7.2	26.6

Abbreviations: MD, mean diameter; PanCan, Pan-Canadian Early Detection of Lung Cancer Study; PKUPH, Peking University People’s Hospital; UKLS, UK Lung Cancer Screening; VA, US Department of Veterans Affairs; VOL, volume.

^a^ Models were applied to the low-dose computed tomography image on which nodules were first seen.

^b^ Nodule size is defined as largest diameter in millimeters.

^c^ Model fitted on screening data.

^d^ Model fitted on data from a clinical setting.

Table 4 shows estimated sensitivity, specificity, and positive and negative predictive values for malignancy at 2%, 5% or 10% model probability thresholds for nodules observed in the prevalence screening for the PanCan and UKLS models. PanCan-1b yielded highest sensitivities at 2% risk threshold (0.81 [95% CI, 0.67-0.94]), and 10% risk threshold (0.52 [95% CI, 0.32-0.71]), but lower specificities (2%: 0.90 [95% CI, 0.89-0.92]; 10%: 0.99 [95% CI, 0.98-0.99]) than the other models. The highest specificities at the 2% and 10% risk thresholds were observed for PanCan-MD (2%: 0.96 [95% CI, 0.95-0.97]; 10%: 0.99 [95% CI, 0.99-1.00]) but were accompanied by lower sensitivities (2%: 0.72 [95% CI, 0.56-0.86]; 10%: 0.43 [95% CI, 0.25-0.61]). The lowest positive predictive values at 2% or 5% risk thresholds were for PanCan-1b (0.08 [95% CI, 0.05-0.12]; 5%: 0.19 [95% CI, 0.11-0.27]), while the highest were for PanCan-MD (2%: 0.17 [95% CI, 0.10-0.24]; 5%: 0.33 [95% CI, 0.20-0.45]). On our data, PanCan-1b showed very similar estimates for sensitivity and specificity compared with those from the original PanCan study data.^15^ However, the recent PanCan-VOL and PanCan-MD updates in PanCan showed higher sensitivity and lower specificity in our study but similar PPV.^16^ External validation of PanCan-2b in the NLST also showed higher sensitivity, lower specificity, and comparable PPV^18^ compared with estimates in LUSI (Table 4). The UKLS model showed inferior sensitivity (2%: 0.25 [95% CI, 0.11-0.39]; 5%: 0.20 [95% CI, 0.07-0.33]; 10%: 0.14 [95% CI, 0.01-0.27]) and specificity (2%: 0.83 [95% CI, 0.81-0.85]; 5%: 0.92 [95% CI, 0.91-0.94]; 10%: 0.97 [95% CI, 0.96-0.98]) compared with PanCan models.

**Table 4.** Classification Performance at Predicted Risk Thresholds in LUSI During Prevalence Screenings and Other Screening Studies

Model	Threshold of Predicted Risk (95% CI)		
	0.02	0.05	0.10
LUSI			
PanCan-1b			
Sensitivity	0.81 (0.67-0.94)	0.72 (0.56-0.87)	0.52 (0.32-0.71)
Specificity	0.90 (0.89-0.92)	0.97 (0.96-0.98)	0.99 (0.98-0.99)
PPV	0.08 (0.05-0.12)	0.19 (0.11-0.27)	0.29 (0.16-0.42)
NPV	1.00 (1.00-1.00)	1.00 (0.99-1.00)	0.99 (0.99-1.00)
PanCan-2b			
Sensitivity	0.79 (0.65-0.92)	0.69 (0.51-0.86)	0.50 (0.32-0.67)
Specificity	0.92 (0.91-0.94)	0.97 (0.96-0.98)	0.99 (0.99-1.00)
PPV	0.11 (0.07-0.16)	0.23 (0.14-0.32)	0.39 (0.24-0.55)
NPV	1.00 (1.00-1.00)	1.00 (0.99-1.00)	0.99 (0.99-1.00)
PanCan-MD			
Sensitivity	0.72 (0.56-0.86)	0.66 (0.48-0.84)	0.43 (0.25-0.61)
Specificity	0.96 (0.95-0.97)	0.98 (0.98-0.99)	0.99 (0.99-1.00)
PPV	0.17 (0.10-0.24)	0.33 (0.20-0.45)	0.36 (0.19-0.52)
NPV	1.00 (0.99-1.00)	1.00 (0.99-1.00)	0.99 (0.99-1.00)
PanCan-VOL			
Sensitivity	0.74 (0.60-0.88)	0.56 (0.37-0.74)	0.44 (0.26-0.61)
Specificity	0.94 (0.93-0.95)	0.98 (0.97-0.98)	0.99 (0.99-0.99)
PPV	0.146 (0.08-0.19)	0.24 (0.13-0.34)	0.35 (0.19-0.51)
NPV	1.00 (1.00-1.00)	1.00 (0.99-1.00)	0.99 (0.99-1.00)
UKLS			
Sensitivity	0.25 (0.11-0.39)	0.20 (0.07-0.33)	0.14 (0.01-0.27)
Specificity	0.83 (0.81-0.85)	0.92 (0.91-0.94)	0.97 (0.96-0.98)
PPV	0.02 (0-0.03)	0.02 (0-0.04)	0.04 (0-0.08)
NPV	0.99 (0.99-0.99)	0.99 (0.99-0.99)	0.99 (0.99-0.99)
McWilliams et al^15^ PanCan-1b^a^			
Sensitivity	0.85	0.71	0.60
Specificity	0.90	0.96	0.98
PPV	0.11	0.19	0.25
NPV	1.00	1.00	0.99
Tammemägi et al^16^^a^			
PanCan-MD			
Sensitivity	0.90	0.84	0.70
Specificity	0.87	0.93	0.96
PPV	0.12	0.19	0.28
NPV	1.00	1.00	0.99
PanCan-VOL			
Sensitivity	0.91	0.85	0.69
Specificity	0.87	0.93	0.96
PPV	0.13	0.20	0.28
NPV	1.00	1.00	0.99
White et al^18^ PanCan-2b^a^			
Sensitivity	0.97	0.93	0.85
Specificity	0.77	0.89	0.94
PPV	0.10	0.19	0.27
NPV	1.00	1.00	1.00

Abbreviations: LUSI, Lung Cancer Screening Intervention; MD, mean diameter; NPV, negative predictive value; PanCan, Pan-Canadian Early Detection of Lung Cancer Study; PPV, positive predictive value; VOL, volume.

^a^ Data on 95% CIs not provided in the source articles.

Logistic regression models fitted via generalized estimating equations on LUSI data combined with model selection via backward elimination based on the quasi–Akaike Information Criterion resulted in a model retaining age (β coefficient = 0.06 [95% CI, 0.01 to 0.11]; *P* = .02), years since quitting smoking (β coefficient = 0.83 [95% CI, −0.15 to 1.82]; *P* = .10), bronchitis (β coefficient = −1.23 [95% CI, −2.29 to −0.17]; *P* = .03), nodule mean diameter (β coefficient = 0.14 [95% CI, 0.09 to 0.19]; *P* < .001), nodule location (β coefficient = 1.23 [95% CI, 0.35 to 2.11]; *P* = .01), and spiculation (β coefficient = 1.72 [95% CI, 1.02 to 2.42]; *P* < .001) as significant variables (eTable 8 in the Supplement), whereas sex, self-reported history of extrathoracic cancer, smoking duration, emphysema (based on CT results), FVC, nodule type, and nodule count per scan showed no further association with malignancy. This final model yielded discrimination AUC of 0.90 (95% CI, 0.83-0.93)^24^ (bootstrap AUC, 0.88 [95% CI, 0.84-0.92]) for nodules detected in the prevalence round and 0.81 (95% CI, 0.71-0.90) (bootstrap AUC, 0.81 [95% CI, 0.73-0.87]) for nodules first detected in the incidence round.

## Discussion

Using data of the German LUSI trial, this diagnostic study found that 4 variants of the PanCan prediction model, developed in context of the Canadian Early Detection of Lung Cancer Study, each provided good discrimination between malignant and nonmalignant pulmonary nodules observed at individuals’ first (prevalence) LDCT screening, whereas more moderate discrimination was observed for 3 models originally developed on the basis of patient data from the Mayo Clinic and VA and PKUPH clinics. By contrast, discrimination for the model recently developed in context of the UKLS was poor.

To our knowledge, these analyses are the first to provide external validation of the recent UKLS model^17^ and a complete external validation for PanCan-MD and PanCan-VOL models.^16^ Our analyses showed no clear superiority for the PanCan-VOL or PanCan-MD models over the older PanCan model versions, confirming observations in PanCan^16^ but not those from Horeweg et al.^30^ In LUSI, diameters were retrieved by software, thus based on a 3-dimensional estimation, which possibly led to a more accurate estimation of nodule diameter compared with manual diameter measurements used in other studies.^31^ Overall, our observations are in line with those from other validation studies, in context of incidentally or symptomatically detected pulmonary nodules^13,32,33,34,35,36,37,38^ or in LDCT screening settings,^14,17,18,19,39,40^ which mostly have also shown discrimination indices of AUCs of approximately 0.80 and higher, and similar ranking by discrimination capacity when comparing performance of the PanCan, VA, Mayo Clinic, and PKUPH models.

Aside from good discrimination, PanCan-1b, PanCan-2b, and PanCan-VOL, but not PanCan-MD or UKLS, also showed acceptable calibration of estimated absolute malignancy probabilities when applied to data from the prevalence screening. In the original study leading to development of the initial PanCan models, acceptable calibration was reported on validation on data from a chemoprevention trial by the British Columbia Cancer Agency,^15^ and for PanCan-1b and PanCan-2b in NLST data^14,18^ and a pilot LDCT screening trial in Queensland, Australia.^39,40^ For the more recent PanCan-MD and PanCan-VOL models, by contrast, Tammemägi et al^16^ reported lower calibration accuracy by such tests in NLST data. The PanCan-1b model showed similar sensitivity, specificity, and positive predictive values at selected (2%, 5%, or 10%) probability thresholds in LUSI and PanCan data,^15^ while among LUSI, PanCan, and NLST data, these estimates were somewhat more variable for PanCan-2b, PanCan-VOL, and PanCan-MD.^16,18^ For the models developed in clinical settings, we found overestimated malignancy risk estimates, which were also observed for the VA and Mayo Clinic models on NLST data.^14^ None of the models (including the PanCan variants) showed acceptable calibration when applied to nodules first observed in incidence screens.

Depending on the context (ie, clinical or screening) in which they were developed, malignancy prediction models include different sets of predictor variables. While age is an established factor for lung cancer risk, it was not retained as predictor in most PanCan models (PanCan-1b, PanCan-MD, or PanCan-VOL) or in those fitted in LUSI. However, in the UKLS model, age appeared together with smoking duration. With regard to smoking, while lifetime smoking status (ever vs never) is not relevant in LUSI or other screening contexts, including UKLS and PanCan, as screening generally is targeted to long-term smokers, our data and the analyses for the development of UKLS model^17^ show that in multivariable models that include age and detailed nodule characteristics, additional smoking-related information may improve malignancy prediction in combination with CT imaging, even among individuals at high risk who are eligible for lung cancer screening.

Sex, associated with increased risk of malignancy for women based on the PanCan and UKLS models, showed no predictive value in our data and was found to be associated with lower malignancy risk in the Danish Lung Cancer Screening Trial.^19^ The female-to-male sex ratio for lung cancer incidence varies across populations and by age, in relation to variable prevalence of and changing trends in smoking habits among women,^41^ which could explain population differences in the predictive value of sex for nodule malignancy. Also, among patients with lung cancer, women more often have adenocarcinomas as opposed to other histologic tumor subtypes.^5,42^ Adenocarcinomas develop from slowly growing nodules with longer lead time until clinical relevance, which makes a benefit from lung cancer screening in terms of mortality reduction more likely.^2,5,42^ Thus, it may be relevant to refine malignancy prediction by sex and tailor models to specific screening populations.

The PanCan and UKLS models, which were fitted on data from a first prevalence screening, may be less applicable to new nodules in follow-up screenings.^16^ In NLST, the malignancy risk of nodules 4 to 6 mm or larger or 6 to 8 mm or larger was higher than that of nodules found at the baseline screening, and malignancies associated with new nodules were significantly less likely to be adenocarcinomas.^43^ We could not confirm these observations in LUSI data because of small numbers (eTable 3 in the Supplement).

### Strengths and Limitations

Our study has some strengths, including that (with only 2 exceptions), malignancy was linked to specific nodules, as opposed to NLST in which malignancy was assumed to be always linked the largest nodule observed.^14,16,18^ Also, our study is the first, to our knowledge, to compare and document differences in the performance of malignancy risk predictions for models applied to nodules detected in prevalence or incidence screenings and for models originally developed in screening vs clinical settings.

Our study also has some limitations. One limitation is the missing information in LUSI for several risk factors, including family history of lung or extrathoracic cancer and possible differences in CT-based assessment of emphysema as compared with PanCan.

A limitation of all models examined in this analysis is that they do not incorporate measures of longitudinal nodule growth as a predictor of malignancy. For nodules of intermediate size, current diagnostic criteria for determining malignancy include early recall examinations for the determination of volume doubling time of nodules observed at an individual’s first screening or a direct assessment of longitudinal growth for nodules already noted at earlier visits when screening participants return for annual follow-up screenings. Detection algorithms are now being developed that integrate relevant nodule and nonnodule features on repeated CT screening examinations over time to predict the presence of lung cancer.^30,44^

Variables difficult to standardize across populations, such as lung disease diagnosis and nodule type categories, may hinder model transferability. In fact, a reduced UKLS model (eTable 2 in the Supplement) that ignored variables with definitions or prevalence markedly differing from LUSI performed better in our data compared with the original version. A more general limitation not only of LUSI but also other studies, including PanCan and UKLS, is the relatively small study size, with 2028 participants at baseline screenings in UKLS, 2029 participants at baseline screenings in LUSI, and 2537 participants at baseline screenings in PanCan, and limited numbers of malignant nodules observed. Accuracy of variable selection and model calibration may be improved in data from larger studies (eg, based on pooled data worldwide).

## Conclusions

Our findings suggest that the PanCan models have good discrimination and confirmatory evidence for calibration accuracy of predicted malignancy risks when applied to nodules observed in an individual’s first (prevalence) screening examination, suggesting that such models may become useful tools for optimizing nodule management in population screening settings. Estimates of current models seem most applicable to nodules detected on a first screening examination, the specific context in which PanCan and UKLS were developed. For individuals screened at regular intervals, as in organized screening programs, models may be further developed to incorporate estimates of nodule volume doubling times, determined on early recall follow-up CT or determined directly for nodules already noted on an earlier visit when individuals return for annual incidence screenings.^44^

## Notes

Supplement. **eTable 1.** Low-Dose Computed Tomography Evaluation Algorithm Applied in the German Lung Cancer Screening Intervention Trial **eAppendix.** Supplementary Methods **eTable 2.** Coefficients of the Selected Lung-Cancer Risk Prediction Models **eFigure 1.** Flow Graph Showing Inclusion and Exclusion Criteria for Lung Cancer Screening Intervention Trial Low-Dose Computed Tomography Arm Participants **eTable 3.** Nodule Count By Size, Screening Round, Malignancy Status, and Nodule Type **eFigure 2.** Receiver Operating Characteristic Curves of Nodules First Seen in the Incidence Screening Rounds **eTable 4.** Observed vs Predicted Nodule Malignancy by Nodule Size in the Incidence Screening Rounds **eTable 5.** Observed vs Predicted Nodule Malignancy Rates by Deciles of Predicted Risk in the Prevalence Screening Round **eTable 6.** Observed vs Predicted Nodule Malignancy Rates by Deciles of Predicted Risk in the Incidence Screening Rounds **eTable 7.** Evaluation of Absolute Risk Calibration of the Selected Models by Screening Round **eTable 8.** Coefficients of Multivariable Logistic Regression Models Fitted on Data From the Lung Cancer Screening Intervention Trial
