# Supplementary material for: Evaluation of Prediction Models for Identifying Malignancy in Pulmonary Nodules Detected via Low-Dose Computed Tomography
Source: JAMA Netw Open. 2020 Feb 14;3(2):e1921221. doi: 10.1001/jamanetworkopen.2019.21221 (PMC12543391; doi:10.1001/jamanetworkopen.2019.21221)
Supplement: Supplement. — eTable 1. Low-Dose Computed Tomography Evaluation Algorithm Applied in the German Lung Cancer Screening Intervention Trial eAppendix. Supplementary Methods eTable 2. Coefficients of the Selected Lung-Cancer Risk Prediction Models eFigure 1. Flow Graph Showing Inclusion and Exclusion Criteria for Lung Cancer Screening Intervention Trial Low-Dose Computed Tomography Arm Participants eTable 3. Nodule Count By Size, Screening Round, Malignancy Status, and Nodule Type eFigure 2. Receiver Operating Characteristic Curves of Nodules First Seen in the Incidence Screening Rounds eTable 4. Observed vs Predicted Nodule Malignancy by Nodule Size in the Incidence Screening Rounds eTable 5. Observed vs Predicted Nodule Malignancy Rates by Deciles of Predicted Risk in the Prevalence Screening Round eTable 6. Observed vs Predicted Nodule Malignancy Rates by Deciles of Predicted Risk in the Incidence Screening Rounds eTable 7. Evaluation of Absolute Risk Calibration of the Selected Models by Screening Round eTable 8. Coefficients of Multivariable Logistic Regression Models Fitted on Data From the Lung Cancer Screening Intervention Trial [file jamanetwopen-e1921221-s001.pdf]

Supplementary Online Content

González Maldonado S, Delorme S, Hüsing A, et al. Evaluation of prediction models for identifying malignancy in pulmonary nodules detected via low-dose computed tomography. *JAMA Netw Open*. 2020;3(2)e1921221. doi:10.1001/jamanetworkopen.2019.21221

eTable 1. Low-Dose Computed Tomography Evaluation Algorithm Applied in the German Lung Cancer Screening Intervention Trial

eAppendix. Supplementary Methods

eTable 2. Coefficients of the Selected Lung-Cancer Risk Prediction Models

eFigure 1. Flow Graph Showing Inclusion and Exclusion Criteria for Lung Cancer Screening Intervention Trial Low-Dose Computed Tomography Arm Participants

eTable 3. Nodule Count By Size, Screening Round, Malignancy Status, and Nodule Type

eFigure 2. Receiver Operating Characteristic Curves of Nodules First Seen in the Incidence Screening Rounds

eTable 4. Observed vs Predicted Nodule Malignancy by Nodule Size in the Incidence Screening Rounds

eTable 5. Observed vs Predicted Nodule Malignancy Rates by Deciles of Predicted Risk in the Prevalence Screening Round

eTable 6. Observed vs Predicted Nodule Malignancy Rates by Deciles of Predicted Risk in the Incidence Screening Rounds

eTable 7. Evaluation of Absolute Risk Calibration of the Selected Models by Screening Round

eTable 8. Coefficients of Multivariable Logistic Regression Models Fitted on Data From the Lung Cancer Screening Intervention Trial

This supplementary material has been provided by the authors to give readers additional information about their work.

© 2020 González Maldonado S et al. *JAMA Network Open.*

eTable 1. Low-Dose Computed Tomography Evaluation Algorithm Applied in the German Lung Cancer Screening Intervention Trial

**Newly observed nodules (first screening round or new in subsequent rounds)**		**Known nodules (early recalls or subsequent screening rounds)**	
**Outcome by nodule size**	**Action**	**Outcome by nodule growth**	**Action**
without abnormality or nodules < 5mm	back to routine screening (12 months)	-	-
nodules 5 – 7 mm	early recall (6 months)	> 600 VDT<br><br>400 – 600 VDT D < 7.5 mm<br><br>D ≥ 7.5 mm – 10 mm<br><br>≤ 400 VDT or D > 10 mm	back to routine screening<br><br>early recall (6 months)<br><br>early recall (3 months)<br><br>immediate recall
nodules 8 – 10 mm	early recall (3 months)		
nodules > 10 mm / not highly suspicious<br><br>highly suspicious	early recall (3 months)<br><br>immediate recall	non-malignant<br><br>malignant	back to routine screening<br><br>treatment

Abbreviations: VDT: Volume doubling time; D: diameter

© 2020 González Maldonado S et al. *JAMA Network Open.*

eAppendix. Supplementary Methods

Image acquisition and reading

On both systems (Toshiba 16 row and Siemens 128 row scanner) the scans were obtained with a maximum of 1.6–2 mSv radiation exposure. Images were re-constructed at 1mm slice thickness at 0.8 mm (Toshiba) and 0.7 mm (Siemens) intervals, respectively, using edge-enhancing, lung-specific reconstruction algorithms.

The MEDIAN software derived nodule measures by automatically segmenting nodules in all slices containing each of them, and the generated outlines were manually corrected by the operator to include all parts of the nodule and exclude adjacent structures (e.g., vessel sections) falsely recognized by the software as being part of the nodule. Volume, as well as longest, and transverse diameters in the axial plane were automatically derived from the 3D segmented nodule. The lower size limit for nodule detection was (1mm) (largest diameter).

The YACTA software (v 2.5.4.3) identifies lung voxels with a density ≤ -950 HU, a threshold dynamically adjusted in case the air density (outside the patient and inside the trachea) was different from -1,000 HU, as emphysema. Additionally, total lung volume in inspiration (LV), total volume of lung areas with a density below -950 HU (emphysema volume, EV), and the fraction of emphysema volume in relation to the total lung volume in inspiration in percent (emphysema index, EI) were computed. Mean lung density (MLD) in HU resulted from averaging CT-based density values from all voxels of the entire lung. Finally, the 15th percentile of lung density histogram (15TH) was calculated.

Nodule evaluation and management

For newly detected nodules, in any screening round, participants were: sent back to regular annual screening if nodule diameter <5 mm (or no nodules), or invited for earlier follow-up LDCT after 6 months or 3 months if nodules measured 5-7 mm or 8-10 mm, respectively, or recommended immediate diagnostic work-up if with nodules ≥10 mm. In rounds 2-5 (incidence screens), and based on nodule growth, participants with nodules previously seen were sent to regular annual screening if they showed no growth or volume doubling time (VDT) >600 days, invited to LDCT after 6 months or 3 months respectively if VDT 400 – 600 days and, respectively, diameter <7.5 mm or (c) diameter ≥7.5–10 mm, or recommended immediate work-up if VDT ≤400 days or diameter >10 mm.

Linkage of malignancy to individual nodules was achieved by communicating the exact location of the nodule under suspicion to the thoracic surgeon in the context of a preoperative tumor board, in preparation of a resection via a video-assisted thoracoscopy under general anesthesia.

Statistical Analyses

Regarding risk prediction models fitted using data from the LUSI trial: three initial multivariable logistic regression models were fitted to nodules first observed in any screening round, each of them including all variables as reported on eTable 7 in the Supplement, each with a different definition of nodule size: largest diameter, mean diameter and nodule volume. Those three models were compared with one another based on the quasi-AIC (QIC), with the model including mean diameter yielding the best results (QIC=541.6 vs. 549.9 for largest diameter and 592.2 for nodule volume). With this model as starting point, we performed backward feature elimination based on p-values (>0.05) and at each step ranked the resulting reduced models by their QIC as calculated by the function “model.sel”, available in the MuMIn R package.

© 2020 González Maldonado S et al. *JAMA Network Open.*

eTable 2. Coefficients of the Selected Lung-Cancer Risk Prediction Models

Predictors / variables		PanCan 1b[a]	PanCan 2b[a]	PanCan MD[b]	PanCan VOL[c]	UKLS	Mayo	PKUPH[d]	VA
	**Intercept**	-6.6144	-6.7892	-6.5355	-6.4432	-2.2915	-6.8272	-4.496	-8.404
**Participant-Related Characte-ristics**	**Age (years)**		0.0287			-0.0257	0.0391	0.070	0.078
	**Sex (female vs male)**	0.6467	0.6011	0.3749	0.3642	0.5105			
	**Family history of lung cancer[e] (yes/no)**		0.2961			1.9985			2.061
	**Late onset (>60 y)**					1.5724			
	**History of cancer[f] (excl. lung)**					0.5305	1.3388		
	**Smoking (ever/never)**						0.7917		2.061
	**Smoking cessation (years/10)**								-0.567
	**Smoking duration (years)**					0.0565			
	**Asbestos exposure (yes/no)**					0.5884			
	**Asthma (yes/no)**					-0.7777			
	**Bronchitis (yes/no)**					1.7616			
	**Emphysema (yes/no)**		0.2953	0.2879	0.2536				
	**FVC (L)**					-1.1693			
**Nodule-Related Characte-ristics**	**Nodule size (mm, mm³)**								
	**Largest diameter**	-5.5537	-5.3854				0.1274	0.0676	0.112
	**Mean diameter**			-16.1232					
	**Volume**				-9.2285	0.00082			
	**Nodule Type (ref: solid)**								
	**Non-solid, or GGO[g]**		-0.1276	ref	ref	1.6396			
	**Part-solid**		0.3770	0.7005	0.7439	0.4919			
	**Nodule location (upper / else)**	0.6009	0.6581	0.5029	0.5012	-0.1799	0.7838		
	**Nodule count per scan**		-0.0824	-0.0853	-0.0865				
	**Spiculation (yes/no)**	0.9309	0.7729	0.9699	0.9502		1.0407	0.736	
	**Border (clear/other)**							- 1.408	

Label (caption):

When applying the PanCan models, nodule size was transformed as follows:

© 2020 González Maldonado S et al. *JAMA Network Open.*

a For models 1b and 2b: largest diameter’ = (((largest diameter)/10)^(-0.5))-1.58113883

b For the mean diameter model: mean diameter’ = ln(1/(largest diameter + perpendicular diameter)/2)- 0.6227482239

c For the volume model: nodule volume’ = (ln(nodule volume)/10)^(-0.5)- 1.619158938, and nodule count was centered: Nodule count’= nodule count – 4

d In the PKUPH model nodule diameter was measured in centimeters

e Family history of lung cancer was not available in our data and was therefore ignored in all related models.

f For the Mayo model, history of cancer (excluding lung) refers to those diagnosed more than 5 years before randomization

g GGO = Ground Glass Opacity

Underlined coefficients indicate variables or variable levels excluded in the alternative version of the UKLS model

Abbreviations: MD: mean diameter; VOL: volume; UKLS: United Kingdom Lung Cancer Screening trial; PKUPH: Peking University People´s Hospital; VA: Veterans Affairs; FVC: Forced vital capacity in liters

© 2020 González Maldonado S et al. *JAMA Network Open.*

eFigure 1. Flow Graph Showing Inclusion and Exclusion Criteria for Lung Cancer Screening Intervention Trial Low-Dose Computed Tomography Arm Participants

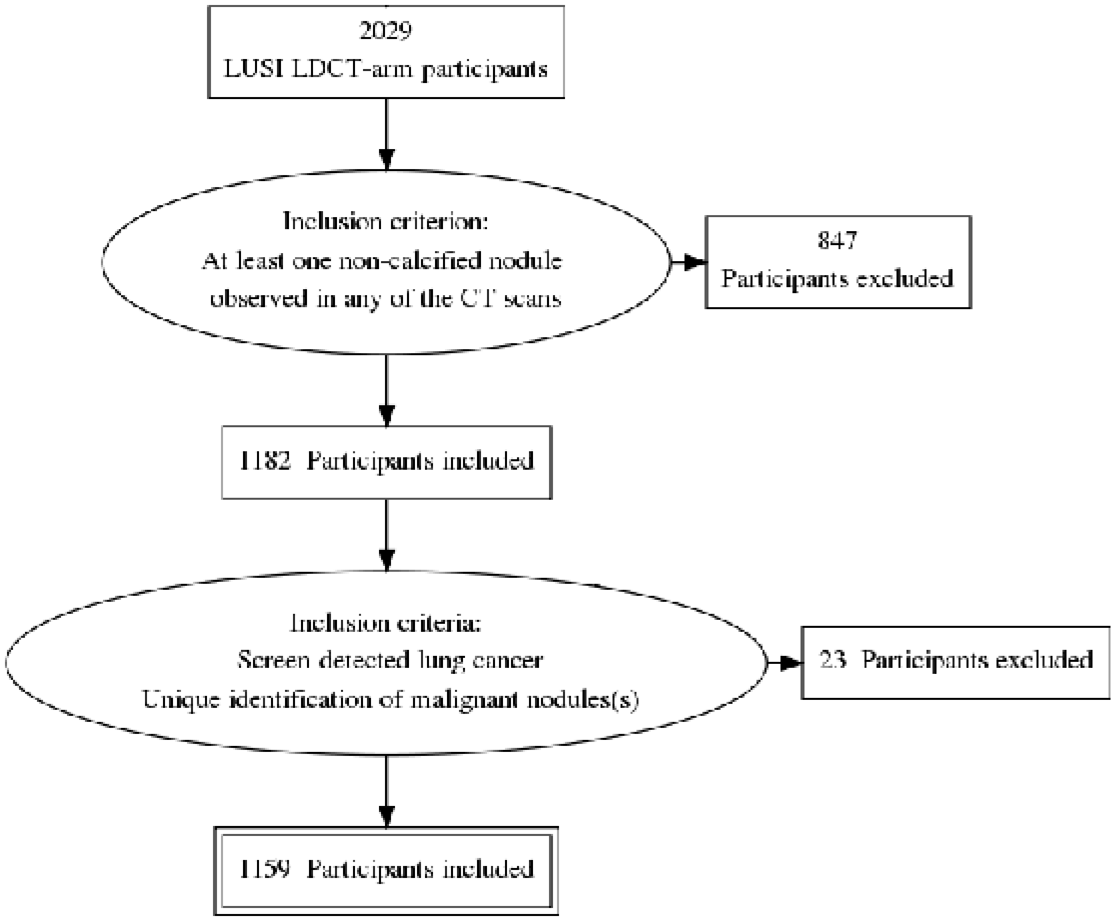

Label (caption):

Screen-detected lung cancer: diagnosis given no more than one year after the last CT scan

© 2020 González Maldonado S et al. *JAMA Network Open.*

eTable 3. Nodule Count By Size, Screening Round, Malignancy Status, and Nodule Type

	First seen on the prevalence round				First seen on any incidence round				First seen on any round			
**Nodule count (%)**	**benign**		**malignant**		**benign**		**malignant**		**benign**		**malignant**	
**Nodule size (largest diameter in mm)**	**solid**	**sub-solid**	**solid**	**sub-solid**	**solid**	**sub-solid**	**solid**	**sub-solid**	**solid**	**sub-solid**	**solid**	**sub-solid**
	2772	79	19	13	875	114	21	10	3647	193	40	23
**<= 3**	213 (7.7)	3 (3.8)	0 (0.0)	0 (0.0)	16 (1.8)	0 (0.0)	0 (0.0)	0 (0.0)	229 (6.3)	3 (1.6)	0 (0.0)	0 (0.0)
**> 3 and <= 4**	1038(37.4)	13 (16.5)	1 (5.3)	0 (0.0)	121(13.8)	1 (0.9)	0 (0.0)	1 (10.0)	1159(31.8)	14 (7.3)	1 (2.5)	1 (4.3)
**> 4 and <= 5**	649 (23.4)	5 (6.3)	1 (5.3)	0 (0.0)	136 (15.5)	10 (8.8)	0 (0.0)	0 (0.0)	785 (21.5)	15 (7.8)	1 (2.5)	0 (0.0)
**> 5 and <= 6**	366 (13.2)	14 (17.7)	2 (10.5)	0 (0.0)	221 (25.3)	19 (16.7)	1 (4.8)	0 (0.0)	587 (16.1)	33 (17.1)	3 (7.5)	0 (0.0)
**> 6 and <= 8**	368 (13.3)	24 (30.4)	5 (26.3)	0 (0.0)	190 (21.7)	16 (14.0)	3 (14.3)	1 (10.0)	558 (15.3)	40 (20.7)	8 (20.0)	1 (4.3)
**> 8 and <= 10**	83 (3.0)	8 (10.1)	2 (10.5)	3 (23.1)	73 (8.3)	17 (14.9)	4 (19.0)	1 (10.0)	156 (4.3)	25 (13.0)	6 (15.0)	4 (17.4)
**> 10**	55 (2.0)	12 (15.2)	8 (42.1)	10 (76.9)	118 (13.5)	51 (44.7)	13 (61.9)	7 (70.0)	173 (4.7)	63 (32.6)	21 (52.5)	17 (73.9)

© 2020 González Maldonado S et al. *JAMA Network Open.*

eFigure 2. Receiver Operating Characteristic Curves of Nodules First Seen in the Incidence Screening Rounds

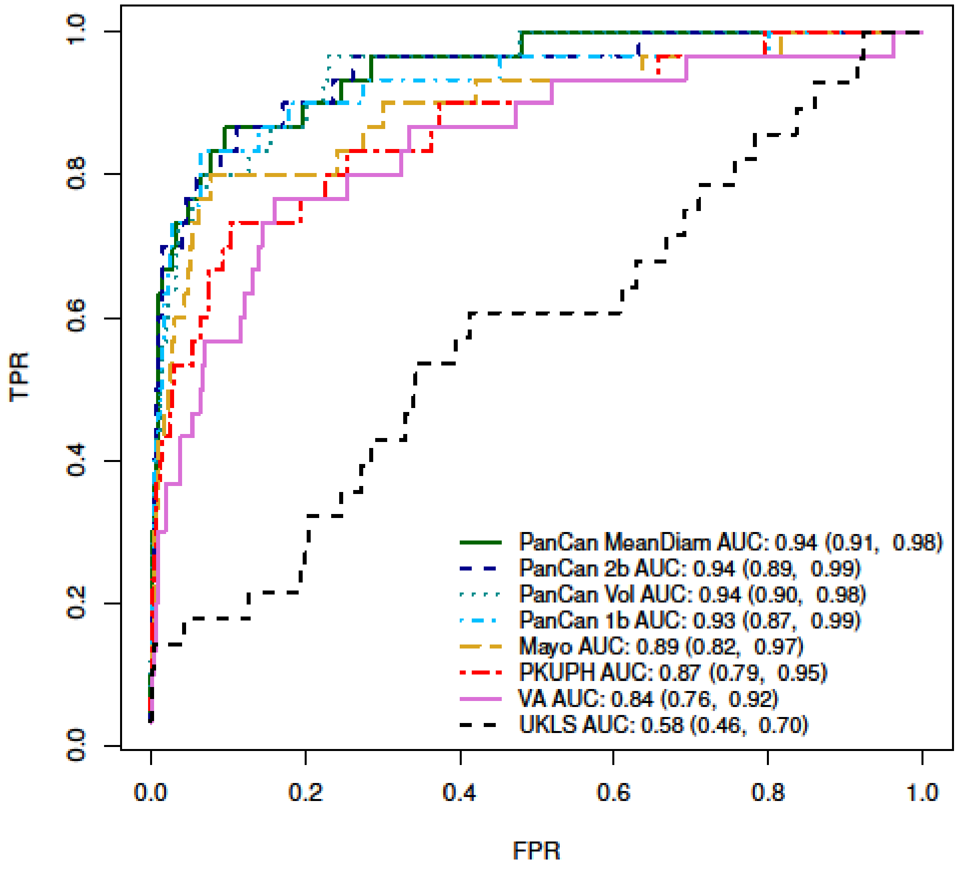

Figure legend (caption):

Areas under the curve are accompanied by 95% confidence intervals

Abbreviations: AUC: Area under the curve, Vol: volume; VA: Veterans Affairs; PKUPH: Peking University People´s Hospital; UKLS: United Kingdom Lung Cancer Screening trial

© 2020 González Maldonado S et al. *JAMA Network Open.*

eTable 4. Observed vs Predicted Nodule Malignancy by Nodule Size in the Incidence Screening Rounds

Nodule size (mm)	Total nodule count	Malignant nodule count	Observed malignancy rate	Models fitted on screening data					Models fitted on data from a clinical setting		
				PanCan 1b	PanCan 2b	PanCan MD	PanCan VOL	UKLS	Mayo	PKUPH	VA
< 5	253	1	0.4%	0.3%	0.3%	0.03%	0.16%	1.1%	6.2%	25.9%	23.1%
5 to < 8	475	5	1.1%	1.4%	1.4%	0.5%	0.9%	1.7%	8.7%	35.4%	27.6%
8 to 10	103	5	4.9%	5.5%	4.9%	2.9%	3.4%	2.3%	14.6%	48.5%	34.3%
> 10	189	20	10.6%	21.7%	18.5%	15.9%	15.2%	10.3%	36.1%	68.6%	57.3%
Total	1020	31	3.0%	5.3%	4.6%	3.5%	3.7%	3.2%	13.7%	40.5%	32.6%

Label (caption):

Nodule size is defined as largest diameter in millimeters.

Abbreviations: MD: mean diameter; VOL: volume; UKLS: United Kingdom Lung Cancer Screening trial; PKUPH: Peking University People´s Hospital; VA: Veterans Affairs

© 2020 González Maldonado S et al. *JAMA Network Open.*

eTable 5. Observed vs Predicted Nodule Malignancy Rates by Deciles of Predicted Risk in the Prevalence Screening Round

Prediction Model	Observed and predicted values	Deciles of predicted nodule malignancy (prevalence round)									
		1st	2nd	3rd	4th	5th	6th	7th	8th	9th	10th
PanCan 1b	Decile cut-offs %	(0.01-0.07)	(0.07-0.12)	(0.12-0.16)	(0.16-0.25)	(0.25-0.32)	(0.32-0.47)	(0.47-0.68)	(0.68-1.1)	(1.1-2.25)	(2.25-79.87)
	Nodule count	318	275	289	281	284	316	256	290	285	289
	Malignant nodule count	0	1	0	0	0	1	0	1	3	25
	Observed malignancy %	0.00	0.36	0.00	0.00	0.00	0.32	0.00	0.34	1.05	8.00
	Predicted malignancy %	0.05	0.10	0.14	0.20	0.28	0.40	0.58	0.87	1.52	8.42
PanCan 2b	Decile cut-offs %	(0.01-0.05)	(0.05-0.09)	(0.09-0.12)	(0.12-0.17)	(0.17-0.24)	(0.24-0.34)	(0.34-0.51)	(0.51-0.83)	(0.83-1.54)	(1.54-68.07)
	Nodule count	289	288	290	286	289	288	288	288	288	289
	Malignant nodule count	0	0	0	1	0	0	0	2	3	26
	Observed malignancy %	0.00	0.00	0.00	0.35	0.00	0.00	0.00	0.69	1.04	9.00
	Predicted malignancy %	0.04	0.07	0.10	0.15	0.20	0.28	0.42	0.65	1.12	6.57
PanCan MD	Decile cut-offs %	(0-0)	(0-0)	(0-0)	(0-0.01)	(0.01-0.02)	(0.02-0.05)	(0.05-0.11)	(0.11-0.26)	(0.26-0.74)	(0.74-70.76)
	Nodule count	290	287	288	288	289	288	288	288	288	289
	Malignant nodule count	0	0	0	0	0	1	0	4	1	26
	Observed malignancy %	0.00	0.00	0.00	0.00	0.00	0.35	0.00	1.39	0.35	9.00
	Predicted malignancy %	0.00	0.00	0.00	0.01	0.02	0.03	0.07	0.18	0.45	5.16
PanCan VOL	Decile cut-offs %	(0-0.02)	(0.02-0.04)	(0.04-0.06)	(0.06-0.09)	(0.09-0.13)	(0.13-0.2)	(0.2-0.31)	(0.31-0.54)	(0.54-1.15)	(1.15-61.11)
	Nodule count	289	288	288	288	289	288	288	288	288	289
	Malignant nodule count	0	0	0	0	0	1	0	3	3	25
	Observed malignancy %	0.0	0.0	0.0	0.0	0.0	0.4	0.0	1.0	1.0	8.7
	Predicted malignancy %	0.0	0.0	0.1	0.1	0.1	0.2	0.3	0.4	0.8	5.5

© 2020 González Maldonado S et al. *JAMA Network Open.*

Prediction Model	Observed and predicted values	Deciles of predicted nodule malignancy (prevalence round) - continued									
		1st	2nd	3rd	4th	5th	6th	7th	8th	9th	10th
UKLS	Decile cut-offs %	(0-0.06)	(0.06-0.1)	(0.1-0.15)	(0.15-0.22)	(0.22-0.36)	(0.36-0.61)	(0.61-1.14)	(1.14-1.94)	(1.94-4.36)	(4.36-100)
	Nodule count	281	280	281	280	281	280	280	281	280	281
	Malignant nodule count	2	2	4	5	0	1	4	4	3	5
	Observed malignancy %	0.71	0.71	1.42	1.79	0.00	0.36	1.43	1.42	1.07	1.78
	Predicted malignancy %	0.04	0.08	0.13	0.18	0.28	0.46	0.81	1.52	2.94	12.67
Mayo	Decile cut-offs %	(2.5-3.3)	(3.3-3.72)	(3.72-4.18)	(4.18-4.8)	(4.8-5.7)	(5.7-6.66)	(6.66-7.7)	(7.7-9.08)	(9.08-11.05)	(11.05-100)
	Nodule count	290	287	288	288	289	288	288	288	288	289
	Malignant nodule count	0	1	0	1	0	1	1	2	0	26
	Observed malignancy %	0.00	0.35	0.00	0.35	0.00	0.35	0.35	0.69	0.00	9.00
	Predicted malignancy %	3.02	3.52	3.96	4.49	5.20	6.19	7.17	8.36	9.90	20.22
PKUPH	Decile cut-offs %	(10.55-12.91)	(12.91-14.26)	(14.26-16.27)	(16.27-18.42)	(18.42-20.83)	(20.83-24.18)	(24.18-28.35)	(28.35-37.28)	(37.28-52.47)	(52.47-99.92)
	Nodule count	289	288	288	288	290	287	288	288	288	289
	Malignant nodule count	0	0	1	1	1	0	3	3	3	20
	Observed malignancy %	0.00	0.00	0.35	0.35	0.34	0.00	1.04	1.04	1.04	6.92
	Predicted malignancy %	12.17	13.60	15.21	17.33	19.67	22.43	26.29	32.03	44.00	63.63
VA	Decile cut-offs %	(8.24-13.79)	(13.79-15.32)	(15.32-16.74)	(16.74-18.43)	(18.43-20.3)	(20.3-22.67)	(22.67-25.79)	(25.79-29.32)	(29.32-34.62)	(34.62-100)
	Nodule count	289	288	288	289	289	287	288	288	290	287
	Malignant nodule count	1	0	0	1	1	1	3	1	6	18
	Observed malignancy %	0.35	0.00	0.00	0.35	0.35	0.35	1.04	0.35	2.07	6.27
	Predicted malignancy %	12.17	14.60	16.06	17.58	19.34	21.44	24.11	27.50	31.91	42.96

Models were applied to the low-dose computed tomography image where nodules were first seen.

Abbreviations: MD: mean diameter; VOL: volume; UKLS: United Kingdom Lung Cancer Screening trial; PKUPH: Peking University People´s Hospital; VA: Veterans Affairs

© 2020 González Maldonado S et al. *JAMA Network Open.*

eTable 6. Observed vs Predicted Nodule Malignancy Rates by Deciles of Predicted Risk in the Incidence Screening Rounds

Prediction Model	Observed and predicted values	Deciles of predicted nodule malignancy (incidence rounds)									
		1st	2nd	3rd	4th	5th	6th	7th	8th	9th	10th
PanCan 1b	Decile cut-offs %	(0.01-0.16)	(0.16-0.37)	(0.37-0.58)	(0.58-0.82)	(0.82-1.16)	(1.16-1.74)	(1.74-2.92)	(2.92-6.4)	(6.4-14.86)	(14.86-82.52)
	Nodule count	102	112	93	101	104	100	102	102	103	101
	Malignant nodule count	0	0	0	2	2	0	4	5	5	13
	Observed malignancy %	0.00%	0.00%	0.00%	1.98%	1.92%	0.00%	3.92%	4.90%	4.85%	12.87%
	Predicted malignancy %	0.09%	0.27%	0.47%	0.70%	0.98%	1.43%	2.24%	4.47%	9.66%	33.09%
PanCan 2b	Decile cut-offs %	(0-0.13)	(0.13-0.32)	(0.32-0.55)	(0.55-0.79)	(0.79-1.16)	(1.16-1.63)	(1.63-2.62)	(2.62-5.26)	(5.26-12.74)	(12.74-81.97)
	Nodule count	102	102	102	102	102	102	102	102	102	102
	Malignant nodule count	0	0	0	1	1	0	3	6	10	10
	Observed malignancy %	0.00%	0.00%	0.00%	0.98%	0.98%	0.00%	2.94%	5.88%	9.80%	9.80%
	Predicted malignancy %	0.07%	0.22%	0.43%	0.66%	0.95%	1.39%	2.12%	3.76%	8.17%	28.63%
PanCan MD	Decile cut-offs %	(0-0)	(0-0.04)	(0.04-0.09)	(0.09-0.17)	(0.17-0.31)	(0.31-0.53)	(0.53-1.26)	(1.26-3.23)	(3.23-9.77)	(9.77-78.07)
	Nodule count	102	102	102	102	102	102	102	102	102	102
	Malignant nodule count	1	0	0	0	0	2	1	8	9	10
	Observed malignancy %	0.98%	0.00%	0.00%	0.00%	0.00%	1.96%	0.98%	7.84%	8.82%	9.80%
	Predicted malignancy %	0.00%	0.02%	0.06%	0.13%	0.24%	0.41%	0.84%	2.04%	5.39%	25.89%
PanCan VOL	Decile cut-offs %	(0-0.05)	(0.05-0.14)	(0.14-0.26)	(0.26-0.42)	(0.42-0.61)	(0.61-0.94)	(0.94-1.71)	(1.71-3.44)	(3.44-9.76)	(9.76-71.91)
	Nodule count	102	102	102	102	102	102	102	102	102	102
	Malignant nodule count	0	0	1	0	1	3	2	6	6	12
	Observed malignancy %	0.00%	0.00%	0.98%	0.00%	0.98%	2.94%	1.96%	5.88%	5.88%	11.76%
	Predicted malignancy %	0.03%	0.09%	0.20%	0.34%	0.51%	0.75%	1.25%	2.45%	5.50%	25.09%

© 2020 González Maldonado S et al. *JAMA Network Open.*

Prediction Model	Observed and predicted values	Deciles of predicted nodule malignancy (incidence rounds) - continued									
		1st	2nd	3rd	4th	5th	6th	7th	8th	9th	10th
**UKLS**	**Decile cut-offs %**	(0.01-0.07)	(0.07-0.13)	(0.13-0.18)	(0.18-0.3)	(0.3-0.48)	(0.48-0.78)	(0.78-1.4)	(1.4-2.55)	(2.55-6.51)	(6.51-100)
	**Nodule count**	100	99	99	99	100	99	99	99	99	100
	**Malignant nodule count**	0	2	4	5	2	3	1	4	5	5
	**Observed malignancy %**	0.00%	2.02%	4.04%	5.05%	2.00%	3.03%	1.01%	4.04%	5.05%	5.00%
	**Predicted malignancy %**	0.04%	0.10%	0.15%	0.23%	0.37%	0.64%	1.03%	1.86%	3.89%	25.29%
**Mayo**	**Decile cut-offs %**	(2.71-4.02)	(4.02-4.8)	(4.8-5.77)	(5.77-6.95)	(6.95-8.28)	(8.28-9.79)	(9.79-12.4)	(12.4-16.72)	(16.72-29.15)	(29.15-99.87)
	**Nodule count**	102	102	101	102	101	102	101	102	101	102
	**Malignant nodule count**	0	1	0	2	0	2	2	4	6	14
	**Observed malignancy %**	0.00%	0.98%	0.00%	1.96%	0.00%	1.96%	1.98%	3.92%	5.94%	13.73%
	**Predicted malignancy %**	3.55%	4.37%	5.29%	6.32%	7.64%	9.04%	10.91%	14.16%	21.70%	54.07%
**PKUPH**	**Decile cut-offs %**	(11.54-15.81)	(15.81-17.93)	(17.93-21.67)	(21.67-25.69)	(25.69-32.11)	(32.11-40.21)	(40.21-55.03)	(55.03-66.37)	(66.37-77.76)	(77.76-99.46)
	**Nodule count**	102	102	102	102	102	102	102	102	102	102
	**Malignant nodule count**	0	0	1	1	1	4	7	1	7	9
	**Observed malignancy %**	0.00%	0.00%	0.98%	0.98%	0.98%	3.92%	6.86%	0.98%	6.86%	8.82%
	**Predicted malignancy %**	14.18%	16.73%	19.88%	23.47%	28.55%	36.30%	47.59%	60.99%	71.38%	86.04%
**VA**	**Decile cut-offs %**	(9.07-16.71)	(16.71-19.07)	(19.07-21.24)	(21.24-24.82)	(24.82-28.01)	(28.01-32.06)	(32.06-36.49)	(36.49-43.3)	(43.3-54.58)	(54.58-99.77)
	**Nodule count**	102	102	101	102	101	102	101	102	101	102
	**Malignant nodule count**	0	0	0	1	3	1	2	4	6	14
	**Observed malignancy %**	0.00%	0.00%	0.00%	0.98%	2.97%	0.98%	1.98%	3.92%	5.94%	13.73%
	**Predicted malignancy %**	14.22%	18.00%	20.06%	22.85%	26.30%	30.03%	34.23%	39.74%	48.35%	72.26%

Models were applied to the low-dose computed tomography image where nodules were first seen.

Abbreviations: MD: mean diameter; VOL: volume; UKLS: United Kingdom Lung Cancer Screening trial; PKUPH: Peking University People´s Hospital; VA: Veterans Affairs

© 2020 González Maldonado S et al. *JAMA Network Open.*

eTable 7. Evaluation of Absolute Risk Calibration of the Selected Models by Screening Round

	Estimate, Test Statistic or p-value	Risk Prediction Model							
		PanCan 1b	PanCan 2b	PanCan MD	PanCan VOL	UKLS	Mayo	PKUPH	VA
**Prevalence round**	**H-L Stat**	7.71	7.23	30.53	10.89	158.99	162.32	1119.9	774.38
	**H-L Test p**	0.56	0.61	<0.001	0.28	<0.001	<0.001	<0.001	<0.001
	**BS all**	0.009	0.009	0.009	0.009	0.012	0.014	0.094	0.063
	**Sp. Z-Stat**	-1.081	0.436	3.888	1.978	-1.076	-12.63	-19.35	-25.24
	**Sp. Test p**	0.28	0.67	<0.001	0.05	0.28	<0.001	<0.001	<0.001
**Incidence rounds**	**H-L Stat**	20.67	12.72	71.08	17.62	283.03	114.56	992.27	478.43
	**H-L Test p**	<0.001	0.01	<0.001	<0.001	<0.001	<0.001	<0.001	<0.001
	**BS all**	0.034	0.034	0.033	0.032	0.041	0.053	0.214	0.134
	**Sp. Z-Stat**	-1.114	-0.277	2.123	1.273	7.09	-6.052	4.925	-9.303
	**Sp. Test p**	0.27	0.78	0.03	0.20	<0.001	<0.001	<0.001	<0.001

Label (caption):

Models were applied to the low-dose computed tomography image where nodules were first seen.

Abbreviations: H-L: Hosmer-Lemeshow; BS: Brier Score; Sp. Z-Stat: Spiegelhalter Z-Statistic; Sp.Test p: Spiegelhalter Z-Test p-value; MD: mean diameter; VOL: volume; UKLS: United Kingdom Lung Cancer Screening trial; PKUPH: Peking University People´s Hospital; VA: Veterans Affairs

© 2020 González Maldonado S et al. *JAMA Network Open.*

eTable 8. Coefficients of Multivariable Logistic Regression Models Fitted on Data From the Lung Cancer Screening Intervention Trial

	All factors included			After variable selection		
**Covariate**	β-coef	95% CI	p-value	β-coef	95% CI	p-value
**Intercept**	-8.27	(-12.16, -4.38)	<0.001	-9.88	(-13.03, -6.73)	<0.001
**Age**	0.07	(0.003, 0.14)	0.05	0.06	(0.01, 0.11)	0.02
**Sex**	-0.23	(-1.07, 0.60)	0.59			
**History of cancer (excl. thorax)**	0.04	(-0.89, 0.97)	0.94			
**Years since quit smoking**	0.74	(-0.19, 1.68)	0.13	0.83	(-0.15, 1.82)	0.10
**Smoking duration (years)**	-0.03	(-0.11, 0.04)	0.39			
**Emphysema**	0.23	(-0.41, 0.88)	0.49			
**Bronchitis**	-1.22	(-2.34, -0.11)	0.04	-1.23	(-2.29, -0.17)	0.03
**FVC**	-0.18	(-0.60, 0.24)	0.41			
**Nodule size (MD, mm)[a]**	0.14	(0.09, 0.19)	<0.001	0.14	(0.09, 0.19)	<0.001
**Nodule type**	-0.58	(-1.67, 0.50)	0.30			
**Nodule location (upper vs middle-or-lower)**	1.23	(0.39, 2.07)	0.004	1.23	(0.35, 2.11)	0.01
**Nodule count per scan**	-0.06	(-0.18, 0.06)	0.34			
**Nodule spiculation**	1.64	(0.93, 2.35)	<0.001	1.72	(1.02, 2.42)	<0.001

[a]MD = mean diameter = (largest diameter + perpendicular diameter)/2

© 2020 González Maldonado S et al. *JAMA Network Open.*
